# Detection of Endometriosis Lesions Using Gd-Based Collagen I Targeting Probe in Murine Models of Endometriosis

**DOI:** 10.1007/s11307-023-01833-6

**Published:** 2023-07-07

**Authors:** Nazanin Talebloo, Maria Ariadna Ochoa Bernal, Elizabeth Kenyon, Christiane L. Mallett, Asgerally Fazleabas, Anna Moore

**Affiliations:** 1 Precision Health Program, Michigan State University, 766 Service Road, East Lansing, MI 48824 USA; 2 Department of Chemistry, College of Natural Sciences, Michigan State University, 578 S Shaw Lane, East Lansing, MI 48824 USA; 3 Department of Obstetrics, Gynecology & Reproductive Biology, Michigan State University, 400 Monroe Avenue NW, Grand Rapids, MI 49503 USA; 4 Department of Animal Science, Michigan State University, 474 S Shaw Ln, East Lansing, MI 48824 USA; 5 Department of Radiology, College of Human Medicine, Michigan State University, East Lansing, MI 48824 USA; 6 Institute for Quantitative Health Science and Engineering, Michigan State University, 775 Woodlot Drive, East Lansing, MI 48824 USA

**Keywords:** Endometriosis, Lesions, Fibrosis, Collagen type I, Gadolinium, Contrast agent, Magnetic resonance imaging

## Abstract

**Purpose:**

Endometriosis is a chronic condition characterized by high fibrotic content and affecting about 10% of women during their reproductive years. Yet, no clinically approved agents are available for non-invasive endometriosis detection. The purpose of this study was to investigate the utility of a gadolinium-based collagen type I targeting probe (EP-3533) to non-invasively detect endometriotic lesions using magnetic resonance imaging (MRI). Previously, this probe has been used for detection and staging of fibrotic lesions in the liver, lung, heart, and cancer. In this study we evaluate the potential of EP-3533 for detecting endometriosis in two murine models and compare it with a non-binding isomer (EP-3612).

**Procedures:**

For imaging, we utilized two GFP-expressing murine models of endometriosis (suture model and injection model) injected intravenously with EP3533 or EP-33612. Mice were imaged before and after bolus injection of the probes. The dynamic signal enhancement of MR T1 FLASH images was analyzed, normalized, and quantified, and the relative location of lesions was validated through *ex vivo* fluorescence imaging. Subsequently, the harvested lesions were stained for collagen, and their gadolinium content was quantified by inductively coupled plasma optical emission spectrometry (ICP-OES).

**Results:**

We showed that EP-3533 probe increased the signal intensity in T1-weighted images of endometriotic lesions in both models of endometriosis. Such enhancement was not detected in the muscles of the same groups or in endometriotic lesions of mice injected with EP-3612 probe. Consequentially, control tissues had significantly lower gadolinium content, compared to the lesions in experimental groups. Probe accumulation was similar in endometriotic lesions of either model.

**Conclusions:**

This study provides evidence for feasibility of targeting collagen type I in the endometriotic lesions using EP3533 probe. Our future work includes investigation of the utility of this probe for therapeutic delivery in endometriosis to inhibit signaling pathways that cause the disease.

**Supplementary Information:**

The online version contains supplementary material available at 10.1007/s11307-023-01833-6.

## Introduction

Endometriosis is a common chronic, inflammatory, and hormone-dependent gynecological condition which is generally characterized by the presence and growth of ectopic, endometrial-like tissue outside of the uterine cavity. This disease mainly affects about 10% of women during their reproductive years [1, 2]. Endometriosis is associated with a variety of symptoms such as pelvic pain, dysmenorrhea, dyspareunia, pain during exercise, urinary dysfunctions, and related fertility problems. Patients with endometriosis can experience one or more symptoms or they can be completely asymptomatic [3–5]. Distinguishing endometriosis from other conditions such as pelvic inflammatory disease, and irritable bowel syndrome is challenging and represents an unmet clinical need. Inability to make this distinction is one of the reasons behind the diagnostic delay among women suffering from endometriosis which can negatively impact their physical, social, and mental well-being in the long run [6–9].

Endometriotic lesions include endometriomas (endometriotic fluid-filled cysts in the ovaries), all forms of peritoneal lesions (red, black, white, scars, and adhesions), and deep infiltrating lesions (DIE) that include nodules that invade peritoneal organs to a depth of 5 mm or more. Red lesions have higher levels of vascularization, while white lesions are red lesions which have undergone through inflammation and fibrosis processes over time [10, 11]. These lesions can be found mainly in the pelvic area, including ovaries, ligaments, bladder, and peritoneal surfaces which are normally categorized into four stages (minimal to severe) based on the extent of the disease. However, the severity of symptoms does not necessarily correlate with this staging system [1, 12, 13]. Multiple endometriosis classification systems exist which are normally governed by complex factors such as color, depth, anatomical location, and size of the lesions [14, 15].

Diagnostic laparoscopy is the gold standard in endometriosis diagnosis which is normally followed by histological verification. However, since this procedure is invasive, and if there is no intention to remove lesions surgically, other non-invasive diagnostic methods in conjunction with physical examination and assessment of patient’s medical history are preferable [16, 17]. Although developing non-invasive and low-invasive methods to diagnose endometriosis represents a challenge, the diagnostic potential of various genetic tests, biomarkers, and imaging techniques have also been evaluated [12, 18–22]. Imaging techniques that are currently used to diagnose endometriosis include sonography and magnetic resonance imaging (MRI). Ultrasound sonography is the first-line imaging modality in assessment of endometriosis [23, 24]. Transabdominal and transvaginal ultrasounds (TVUS) can give a more detailed image of the anatomy, compared to the initial ultrasonography of pelvis area. While TVUS is a readily available and inexpensive tool, it has limitation in detection of lesions, especially the ones located above the rectosigmoid junction and on the peritoneum. Also, its performance is heavily operator dependent, which presents a significant limitation for precise diagnosis [25, 26]. Despite being more expensive, MRI can be used when ultrasound results are unclear and high spatial resolution is needed [27]. Despite dedicated MRI protocols which include both T1 and T2-weighted sequences, this modality can easily overlook solid masses of endometriotic tissue due to their small and atypical signal character [27–31]. Due to limitations presented by ultrasound and MRI, patients with negative imaging results must still be subjected to laparoscopy to obtain a definite diagnosis [16]. Developing targeted contrast agents for MRI can increase the signal difference between the lesions and surrounding tissues, especially for detection of non-pigmented lesions without intrinsic T1 hyperintense signal [32, 33]. Despite several published studies describing the development of targeted contrast agents for imaging of endometriosis, as of today, no clinically approved agents are available [30, 34, 35]. Availability of such agents would significantly aid in precise diagnosis of this disease including the location of the lesions.

Ectopic endometriotic lesions are wounds undergoing repeated cycles of tissue injury and repair (ReTIAR) stimulating microenvironment-mediated epithelial-mesenchymal transition (EMT), fibroblast-to-myofibroblast transdifferentiation (FMT), smooth muscle metaplasia (SMM), and fibrosis [36–38]. The consistent presence of fibrosis in all lesion forms points to endometriosis as a fibrotic condition characterized by excess deposition of collagens, primarily type I collagen [9, 37–40]. Importantly, it was previously shown that immunoreactivity toward collagen type I in the ectopic lesion sections collected from a mouse model of endometriosis was elevated as the disease progressed [41]. Therefore, the presence of fibrosis in endometriotic lesions makes it an attractive biomarker for targeting by affinity ligands incorporated in contrast agents. Previous studies aimed at imaging fibrosis in other pathologies have identified a peptide directed against human collagen type I using phage display [42]. This collagen type I binding peptide consists of 16 amino acids with 10 of them producing the cyclic part between two cysteines. Biphenylalanine (Bip) at the amidated C terminus of the peptide is believed to increase the collagen type I binding ability, with a *Kd* = 1.8 μM. The peptide was synthesized using conventional solid-phase techniques and functionalized with three Gd (DTPA) moieties through thiourea linkages to improve the sensitivity of the contrast agent (termed EP-3533). Relaxivity of EP-3533 was reported as 16.2 mM^−1^ s^−1^ at 4.7 T MR [42–44]. Also, near the common imaging field of 1.5 T, the relaxivity of EP-3533 was five times higher than Magnevist per Gd atom and 15 times higher per molecule [42]. This probe was previously tested with various field strengths and demonstrated successful targeting of fibrotic content in tissues of various pathological conditions at 4.7 T [42], 3 T [45], and 1.4 T [46, 47]. In previous studies, EP-3533 has been used for detection and staging of fibrotic lesions in animal models of liver fibrosis [47–50], pulmonary fibrosis [51], cardiac fibrosis [43], and pancreatic cancer [40].

In this study, we utilized, for the first time, EP-3533 for detection of fibrotic endometriotic lesions using MRI in two murine models of endometriosis. As a result of our studies, we demonstrated that the accumulation of EP-3533 in fibrotic endometriotic lesions in both suture and injection models was significantly higher, compared to its accumulation in control tissue. Also, the specificity of EP-3533 for endometriotic fibrosis was validated by comparison with а control non-binding isomer (EP-3612).

This study serves as the first demonstration of the utility of EP-3533 for magnetic resonance imaging of endometriotic lesions. We believe that this agent can be further investigated as a potential vehicle for delivery of therapeutics targeting various signaling cascades that initiate endometriosis development [52–55].

## Materials and Methods

### Animal Models of Endometriosis

In this study, we used two murine models of endometriosis that differ in the way the disease was initiated.

For the injection model, 8-week-old female Pgr^*cre/+*^ Rosa26^*mTmG/mTmG*^ mice ([56]; *n* = 5) were treated for 3 days with 17β estradiol (E2, Sigma-Aldrich; 1 mg/mL in oil, 0.1 μg/mouse/day) in order to synchronize the estrus cycle and improve lesion development. After the last E2 injection, endometriosis was induced, as previously described by us [57]. Briefly, the lesions were established by inoculating endometrial tissue into the peritoneal cavity. To access the peritoneal cavity, mice underwent a laparotomy under anesthesia and a midventral incision (1 cm) was performed to expose the uterus and intestine. The left uterine horn was removed, placed in a petri dish with sterile PBS, opened longitudinally, and cut into small fragments. The fragments suspended in 0.5 mL sterile PBS were injected into the peritoneal cavity of the same mouse from which the uterus was taken for an autologous implantation, and the abdominal cavity was gently massaged to disperse the tissue. This unique Pgr^*cre/+*^ Rosa26^*mTmG/mTmG*^ mouse model of induced endometriosis is characterized by the presence of the progesterone receptor (Pgr)-positive cells expressing green fluorescent protein (mGFP), allowing for accurate localization and visualization of the endometriotic lesions using fluorescence imaging [56].

In the suture method, endometriosis was induced in 7-week-old female Pgr^*cre/+*^ Rosa26^*mTmG/+*^ mice (*n* = 6). The induction of a suture endometriosis model consisted of two steps. During the first step, mice were ovariectomized. Briefly, the ovaries were isolated and ligated with sterile absorbable suture. A loop suture was placed between the ovary and the tip of the uterus, and the ovaries were then excised. Wound clips were removed 7–10 days post-surgery, and the mice were allowed to recover for 14 days. Following recovery, the ovariectomized mice were treated for 3 days with 17β Estradiol (E2, 0.1 μg/mouse/day) in order to synchronize the estrus cycle prior to second surgery during which one uterine horn was removed. In order to remove a uterine horn, a midline abdominal incision was made, and the caudal end of the uterine horn near the uterotubal junction was cut and ligated with sterile absorbable suture. The removed uterine horn was opened longitudinally, and tissue samples were obtained using a 2-mm dermal biopsy punch. Three biopsies were sutured to the peritoneal wall (3 in each side, total of 6) using a 7-0 braided silk suture. The muscle layer was closed as one layer, then the skin as a second layer. After this procedure, the abdominal incision was closed. A small subcutaneous incision was made at the nape of the neck to create a pocket for an E2 pellet with an expected release concentration of approximately 0.13 mg/day to provide a controlled source of hormone that promotes lesion growth in the absence of the ovaries. Once the pellet was inserted, a wound clip was placed to close the skin. Wound clips were removed 7-10 days post-surgery.

All animal studies were approved by the Institutional Animal Care and Use Committee at Michigan State University and are in compliance with the National Institutes of Health Guide for the Care and Use of Laboratory Animals.

### Contrast Agent for MR Imaging

EP-3533 and EP-3612 contrast agents were purchased from Collagen Medical (Belmont, MA). EP-3612 has an identical structure to EP-3533, except that one of the cysteine moieties is changed from L-Cys in EP-3533 to D-Cys in EP-3612 **(**structure diagram is shown in Suppl. Fig. 1, see ESM**)**. Systematic replacement of amino acids with their D-amino acid counterparts led to a significant decrease in the activity of the peptide, particularly when cysteine amino acid was substituted. This change in chirality results in > 100-fold loss in collagen affinity and design of the non-binding isomer (EP-3612); however, its relaxivity remains equivalent to EP-3533 [40, 42–44].

### Magnetic Resonance Imaging

*In vivo* MRI was performed on both models of endometriosis on average 2 months post-endometriosis induction. Previously, it was shown that endometriotic lesions exhibit a gradual increase in fibrotic content and reach a highly fibrotic state in 6 weeks [41]. Mice were anesthetized using 1.5 to 3% isoflurane in oxygen, and tail veins were catheterized. Temperature (~35 °C) and breathing were monitored and maintained throughout the experiment (SAII Small Animal Instruments, Inc., Stony Brook, NY). For imaging, mice from both groups were injected intravenously with bolus injections of the targeted EP-3533 probe (10 μmol/kg). Control mice in each group were injected with a non-binding EP-3612 probe (10 μmol/kg). This dosage selection was based on prior studies that effectively utilized EP-3533 to specifically target and evaluate fibrotic conditions [40, 46, 51]. Injections were followed by a 100 μL of saline flush over 30 s. Imaging continued for 55 min post-injection with 1- or 2-min temporal resolution. Images were acquired on a 7 Tesla Biospec 70/30 USR (Bruker, Billerica, MA) using an 86-mm-diameter volume transmit coil and 4 channel surface array receive coil (4 × 4 cm). Fat-suppressed T1-weighted FLASH images were acquired for a total acquisition time of 1 h, with *TR* = 76.2 ms, *TE* = 2.6 ms, *FOV* = 30 × 30 mm, 9 slices, 0.5-mm slice thickness, flip angle 30°, and resolution of 200 × 200 × 500 μm. Images were analyzed using Paravision 360 v3.1 software (Bruker). Regions of interest (ROI) were randomly distributed throughout the lesion linings covering most of the lining area. The ROIs of the muscle tissue (mouse leg skeletal muscle) were used as controls within each group of mice. The percent increase of the signal enhancement was calculated for each time point based on a region of interest and normalized to the baseline.

### *Ex vivo* Fluorescence Optical Imaging

After acquiring MR images, mice were euthanized and *ex vivo* fluorescence optical imaging was performed to confirm the location of GPF-expressing lesions in the peritoneal cavity (IVIS Spectrum, Perkin Elmer, Hopkinton, MA). Image analysis was performed using the Living Image 4.2 software (Perkin Elmer).

### Histology

After *ex vivo* imaging, lesions and control tissues (muscle) were collected and either embedded into optimal cutting temperature compound (OCT, Sakura Finetek, Torrance, CA) and immediately frozen in liquid nitrogen or fixed in 10% formalin (Fisherbrand, Pittsburg, PA) and embedded in paraffin. OCT-embedded tissues were cut into 10-μm sections, fixed, and stained with Masson’s Trichrome Stain Kit (Polysciences, Warrington, PA) in accordance with the manufacturer’s instructions. Masson’s trichrome stain is used to assess the collagen content of tissues by staining collagen fibers in blue, cell nuclei in black, and both muscle and cytoplasm in red. Slides stained for collagen were analyzed with a digital pathology scanner (Aperio Versa automated slide scanning imaging system, Leica Biosystems Imaging, Deer Park, IL). Paraffin-embedded sections were stained with hematoxylin and eosin (H&E; VWR). H&E-stained slides were imaged with light microscopy and analyzed with SPOT 4.0 Advance version software (Diagnostic Instruments, Sterling Heights, MI) for the presence of stroma and glands to validate endometriosis lesions.

### ICP-OES Analysis

To quantitate gadolinium content in the collected lesions and muscle tissues, samples were dried, weighed, and digested with 69% nitric acid (TraceSELECT™, Fluka, USA). Samples were then diluted further to 4% nitric acid and filtered to obtain a solution with no visible debris. Gadolinium content was determined for each sample by inductively coupled plasma optical emission spectrometry (ICP-OES) using a 710-ES spectrometer (Varian, Palo Alto, CA). For all ICP-OES measurements, blank nitric acid, and samples with known Gd concentration as calibration were prepared and tested concurrently with test tissue samples. All measurements were carried out in triplicates, and the data were normalized to the dry tissue weight and reported as mean ± SD.

### Statistical Analysis

All data were represented as mean ± SD. Statistical analysis was performed using a two-tailed Student’s *t*-test. *P* < 0.05 was considered statistically significant.

## Results

### Dynamic EP-3533 Enhanced Magnetic Resonance Imaging

**Fig. 1:**
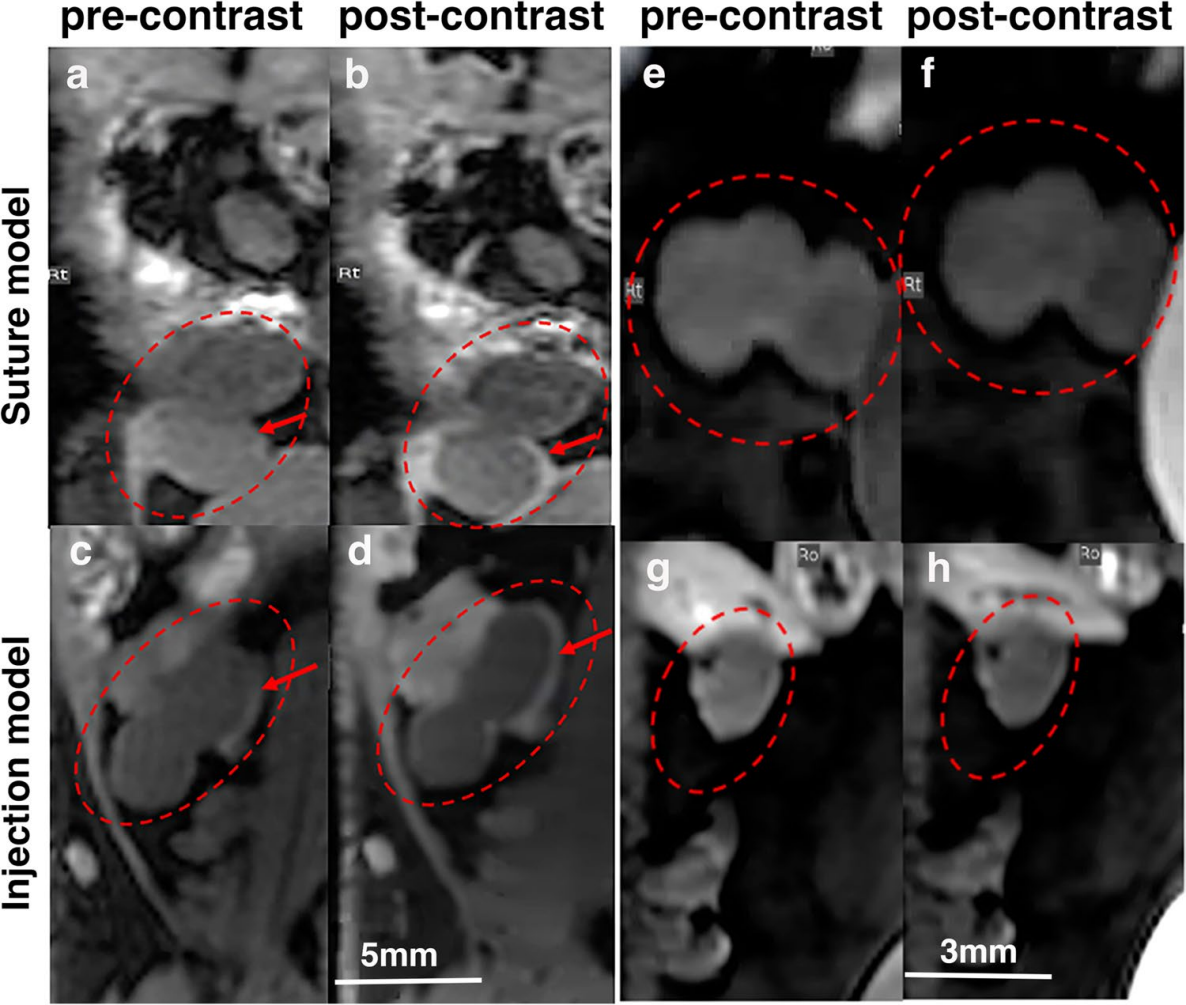
Representative T1-weighted magnetic resonance images of suture and injection mouse models of endometriosis injected with collagen type I targeting probe (EP-3533) or control non-binding probe (EP-3612). Images (**a**), (**b**), (**e**), and (**f**) represent the suture mouse model and images (**c**), (**d**), (**g**), and (**h**) represent the injection mouse model of endometriosis. (**a**) and (**c**) Baseline, before EP-3533 injection and (**b**), (**d**) 55 min post EP-3533 injection. (**e**) and (**g**) Baseline, before EP-3612 and (**f**) and (**h**) 55 min post EP-3612 injection. Red dotted ovals: endometriotic lesions. Red arrows point to endometriotic lesion lining. Note the enhancement of lesion lining 55 min post injection (red arrows) in the group injected with EP-3533. Scale bar for (**a**), (**b**), (**c**), (**d**), (**e**), and (**f**) = 5 mm; scale bar for (**g**) and (**h**) = 3 mm

**Fig. 2:**
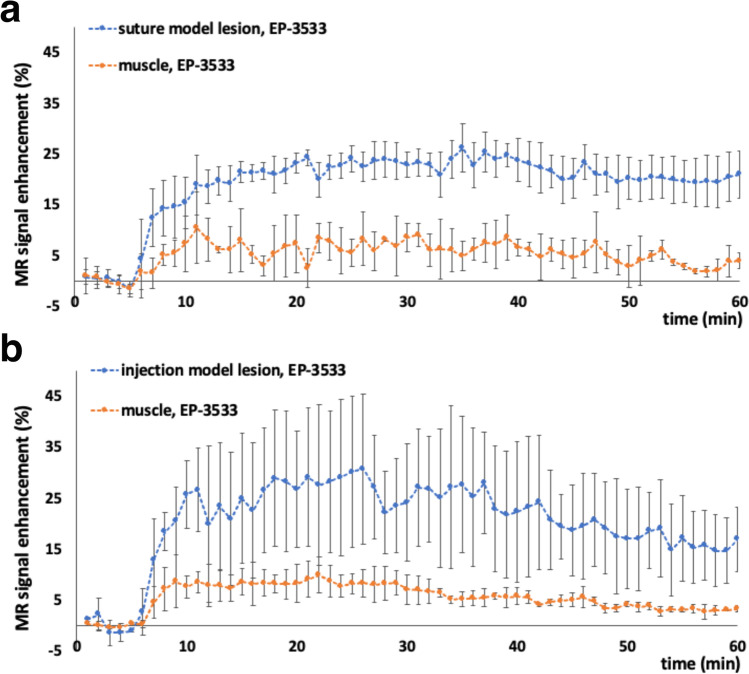
DCE MRI analysis of signal enhancement of endometriotic lesions (blue) and muscle tissue (orange) from mice injected with EP-3533 in **a** suture and **b** injection models. Percentage of MR signal enhancement on *y*-axis was normalized to the average of baseline signal. The probe was injected at 5 min, and mice were imaged for 55 min. Error bars are standard deviations of signal enhancements averaged within the group of mice for each experimental model

To determine whether EP-3533 probe could be used for probing endometriotic lesions based on collagen targeting, we performed dynamic contrast enhanced magnetic resonance imaging (DCE-MRI). We expected that in fibrotic lesions overexpressing collagen, the binding of the contrast agent would lead to a longer signal enhancement and a slower lesion signal washout. As evident from Fig. 1a and c, the endometriotic lesions in both suture and injection models appear relatively homogeneous in the pre-injection T1-weighted MR images. Fifty-five minutes post-EP-3533 injection, there was a relatively rapid signal enhancement in the lesion lining (Fig. 1b and d) during the first 6 min in both suture and injection models which overall led to an average of 21 ± 4.6 and 16.8 ± 6.2% signal enhancement, respectively **(**Fig. 2a and b). The data from the control muscle tissue from both endometriosis models showed a rapid increase (approximately 5–7% signal enhancement) after the injection followed by the washout that did not show an overall significant enhancement 55 min post-injection (3.9 ± 1.5 and 3.3 ± 0.66% for suture and injection models, respectively; Fig. 2a and b).

Comparison of DCE-MR images of control mice pre- (Fig. 1e and g) and post- EP-3612 injection (Fig. 1f and h) showed significantly lower signal enhancement 54 min post-probe injection in both models (0.2 ± 0.5 and 1.9 ± 2.4% signal enhancement for suture and injection models respectively, Suppl. Fig. 2a and b, see ESM), compared to the experimental group injected with EP-3533 in both endometriosis models.

### Lesion Location Validation

**Fig. 3:**
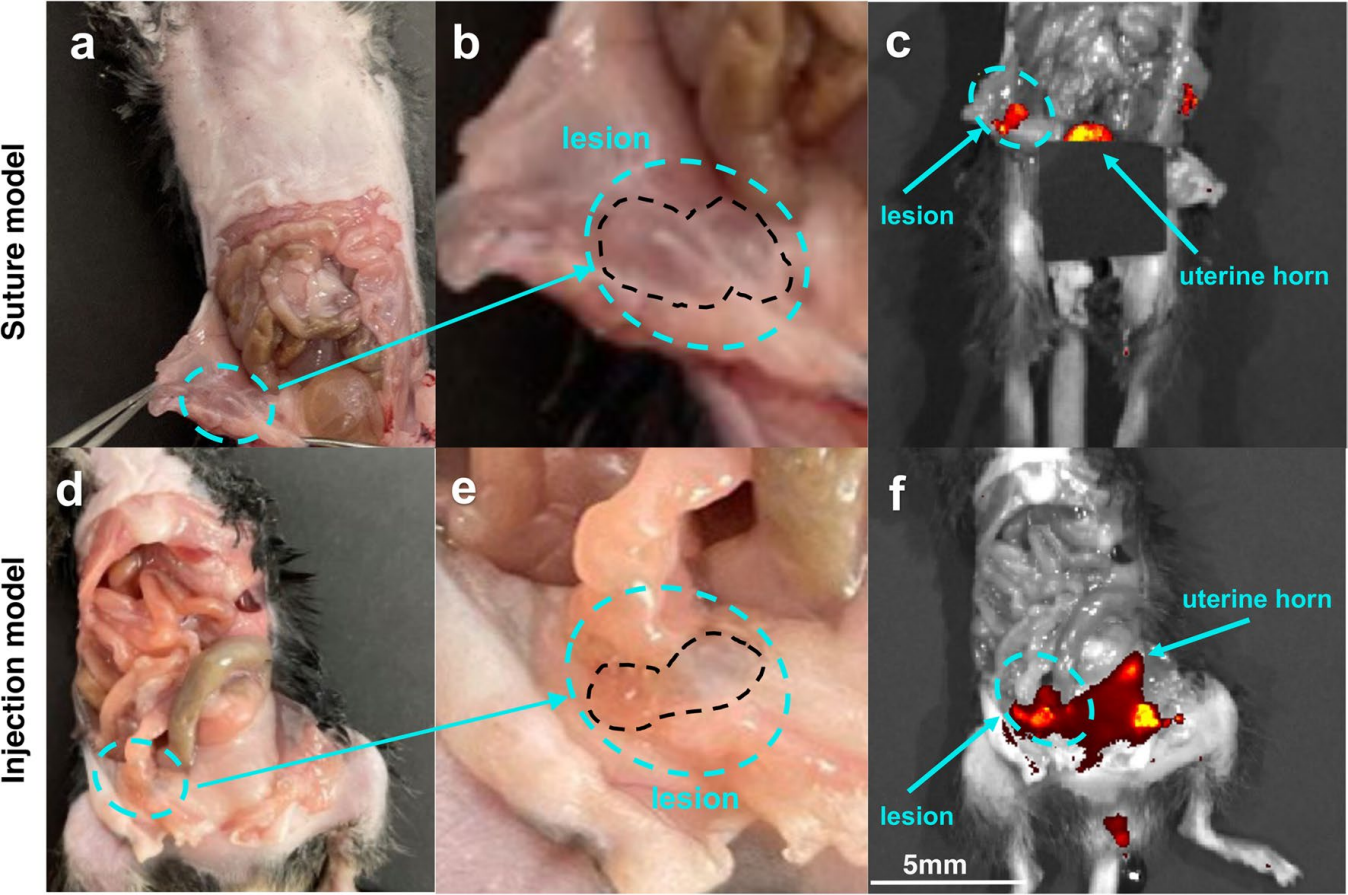
Representative *ex vivo* images of endometriotic lesions. Photographs (**a**) and (**d**) and magnified images of the lesions (**b**) and (**e**) taken after opening the peritoneal wall in suture and injection endometriosis models, respectively. *Ex vivo* fluorescence imaging shows progesterone positive lesions expressing GFP in suture (**c**) and injection (**f**) endometriosis models. Green dotted oval: endometriotic lesions. Black dotted line: border of the lesions. Note that in the injection model (**c**) bladder was covered with black paper to better visualize the signal from the lesions. Scale bar = 5 mm

In order to confirm the presence of the lesions detected with MR imaging, *ex vivo* fluorescence imaging was performed. After the last MR image acquisition, mice were euthanized and the exposed peritoneum was photographed in both suture and injection models (Fig. 3a and d; enlarged images are shown in Fig. 3b and e**)**. To detect and confirm the presence and location of all GPF-expressing lesions, including smaller lesions that might not be easily visible with the naked eye, we performed fluorescence imaging that detected progesterone-positive tissues including uterine horn and endometriotic lesions (Fig. 3c and f**)**. The relative anatomical location of endometriotic lesions observed on MR images aligned with the location of lesions identified using fluorescence imaging. Specific anatomical landmarks in the images are depicted in Suppl. Fig. 3a–h, see ESM. Imaging of excised lesions confirmed GFP expression in both suture and injection models (Suppl. Fig. 4a and b, see ESM). These findings confirmed the presence and location of the lesions that were identified by MRI. To confirm the presence of endometriotic lesions microscopically, collected lesions induced with both suture and injection methods were stained with H&E showing the typical presence of endometrial glands and stroma (Suppl. Fig. 5a and b, see ESM).

### Histological Assessment of Fibrosis

**Fig. 4:**
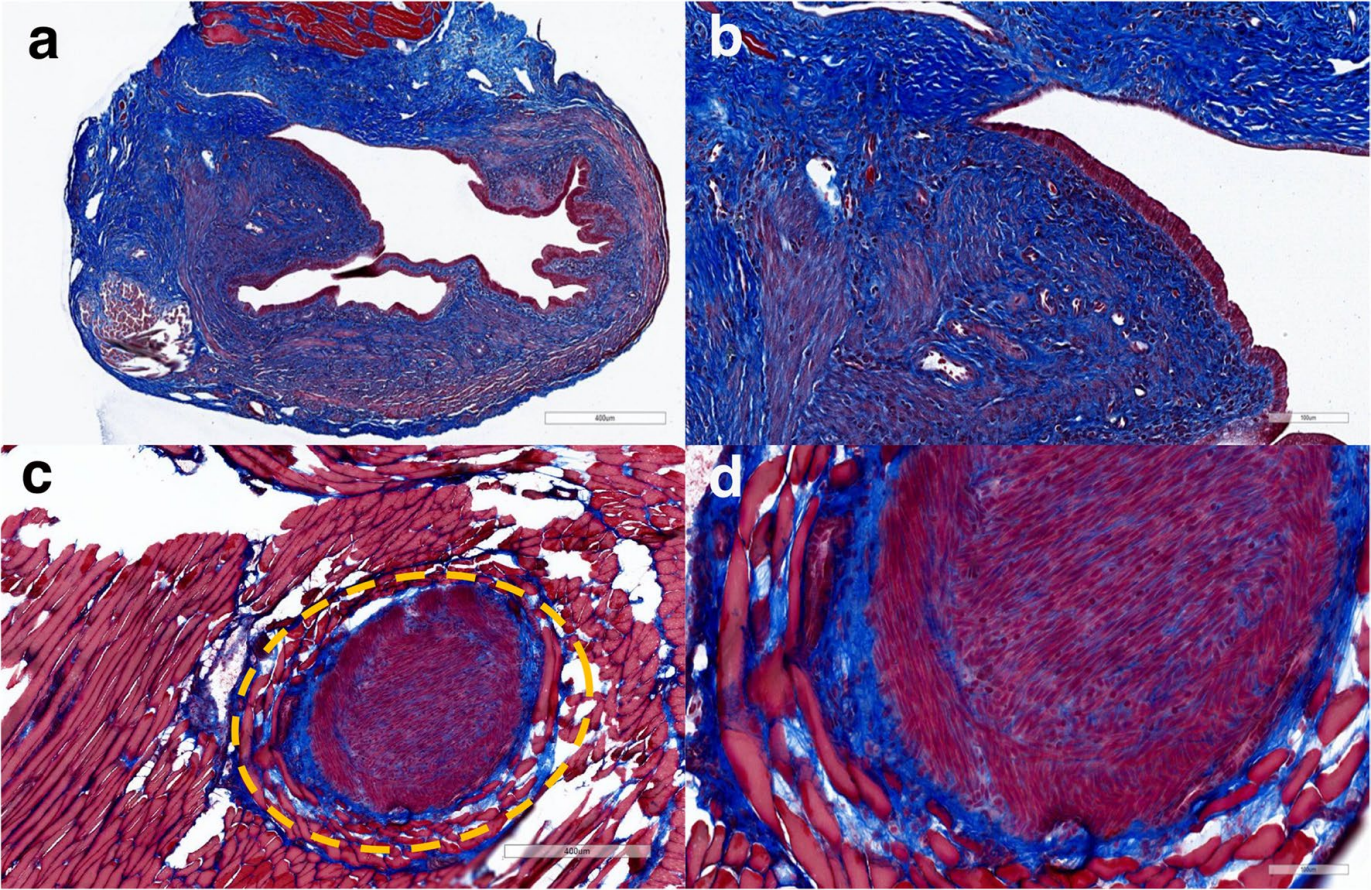
Representative images of Masson trichrome staining of endometriotic lesion tissue sections demonstrating the relative extent of fibrosis. **a**, **b** Lesions from suture model. **c**, **d** Lesion from injection model. Blue — collagen fibers; red — cytoplasm, muscle; black — cell nuclei. Magnification bar = 400 μm (**a**, **c**); 100 μm (**b**, **d**). Lesion in (*c*) is represented by the yellow dotted circle. Note that the collagen content in the suture model is more pronounced than in the injection model

Masson’s trichrome stain is used to assess and visualize the extent of fibrosis in both human biopsies and animal models of human fibrotic disease. Here, we used Masson’s trichrome staining to evaluate the extent of fibrosis in endometriosis mouse models. The results showed a relatively large blue area of the collagen-rich fibrotic regions throughout sections from models induced with either suture (Fig. 4a and b) or injection (Fig. 4c and d) methods, demonstrating overexpression of collagen as a suitable target for EP-3533 probe. It is noteworthy that most of the lesions especially those induced by the suture method were cystic and filled with liquid and showed collagen expression in their lining.

### Quantification of Tissue Gadolinium Content

**Fig. 5:**
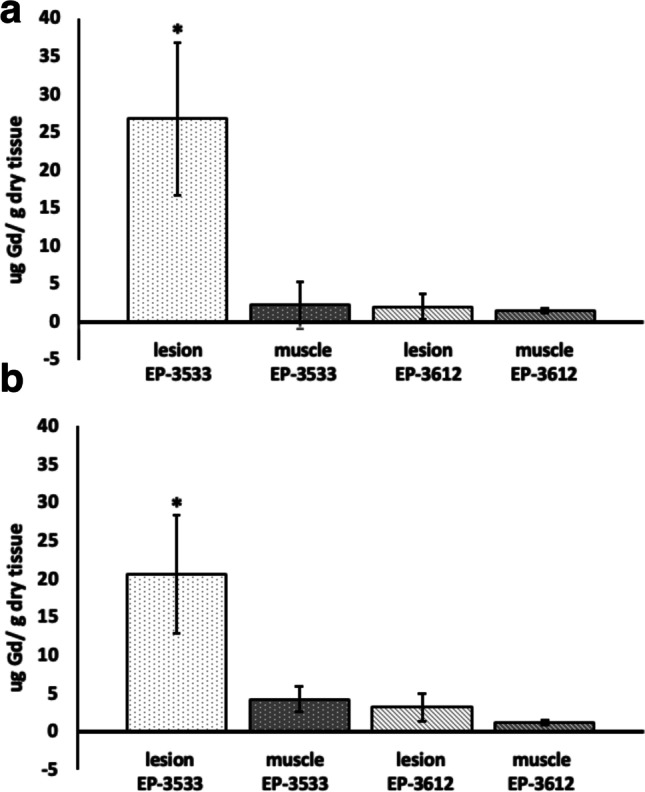
ICP-OES analysis of average gadolinium content in lesion and muscle tissues after injection of EP-3533 and EP-3612 probes in **a** suture and **b** injection models. Gd tissue content was normalized to the dry weight of the tissue. Results are presented as means ± SD. Note that Gd content was significantly higher in endometriotic lesions of mice of both suture and injection models injected with EP-3533, compared to that in the lesions from the animals injected with EP-3612 or in the muscle tissues (*p* < 0.05)

To confirm that the enhancement observed on MR images after the injection of EP-3533 was caused by Gd, we collected tissue samples from both experimental and control groups and analyzed them for Gd content by ICP-OES. The results showed that the Gd concentration in endometriotic lesions of experimental group was 26.7 ± 10.0 and 20.6 ± 7.7μg Gd/g dry tissue in suture and injection models, respectively which was significantly higher than that in skeletal muscle of the same groups (2.2 ± 3.0 and 4.2 ± 1.7 μg Gd/g dry tissue for suture and injection models respectively, *p* < 0.05, Fig. 5a and b). Similarly, injection of a non-binding EP-3612 probe resulted in a low accumulation in the lesions and muscles of both animal models (2 ± 1.6 and 3.2 ± 1.8 μg Gd/g dry lesion tissue and 1.44 ± 0.28 and 1.15 ± 0.27 μg Gd/g dry muscle tissue in suture and injection models respectively, Fig. 5a and b). Our data demonstrate that the only tissues that accumulated a significant amount of Gd were endometriotic lesions from both animal models.

## Discussion

A better understanding of endometriosis is needed to successfully develop new non-invasive diagnostic methods [15] and effective drugs. While imaging is currently used as a non-invasive method to detect endometriotic lesions [23], it does not allow for specific and reliable diagnosis. The main reason is heterogeneity of endometriotic lesions and the paucity of suitable molecular biomarkers. In our search for a consistent biomarker, we focused on a fibrotic aspect of endometriosis and investigated whether imaging probes targeting collagen type I in ectopic endometriotic lesions could be used for its detection. Fibrosis is defined by the overexpression of collagen and is considered as one of the hallmarks of the disease [58, 59]. In this study, a Gd-containing collagen type I binding probe (EP-3533) previously used for imaging fibrosis in multiple animal models [40, 42, 43, 47–51] was evaluated for imaging endometriosis. EP-3612, a non-binding isomer, which only differs from EP-3533 by the chirality of one cysteine residue, thereby significantly reducing its affinity for collagen [42] was used as control.

Recent studies demonstrated that fibrotic component of the lesions, especially collagen type I, tends to increase over time as the disease progresses in both humans and baboon models of endometriosis [36, 39, 41, 60]. Therefore, the rationale behind using the older stage lesion in this proof-of-principle study was based on the fact that higher levels of fibrotic content develop in the later stages of the disease [14]. Since one of the main symptoms of endometriosis is pain, which is often mistaken for menstrual cramps, many patients seek medical advice years after the disease initiation, suggesting a need for a probe which can identify established lesions with significant fibrotic content.

To accomplish this, we used two well-established endometriosis induction methods, namely the suture method and the injection method commonly employed in pre-clinical studies [61]. The primary justification for using both models was based on their application to our studies. While the suture model is not meant to mimic distinct pathologies, it provides for the exact location of the lesions so they can be identifiable in our proof-of-concept imaging studies. Equipped with this knowledge, we next moved to the injection model where the locations of the lesions are random and closely represent a clinical scenario situation. Our studies demonstrated that it was feasible to detect lesions in both models using EP 3533.

The question may be raised whether exogenous estrogen used for the lesion induction could impact collagen type I expression/detection by EP3533. It is noteworthy that mice in both models received estrogen injections for 3 days prior to lesion induction to synchronize the uterus. Following the inoculation, the animals had normal ovarian hormone levels associated with the different stages of the estrous cycle. This mimics the natural hormonal fluctuations experienced by women who are not undergoing any form of suppressive hormone therapy.

Both mouse models of endometriosis were dynamically imaged with MRI T1 FLASH sequence pre- and post-injection of the probes. This was achieved by the acquisition of a series of baseline images without contrast enhancement, followed by a series of images acquired over time during and after the administration of the contrast agent.

In both endometriosis models, MR images showed an increase in signal intensity of lesion lining which appeared brighter compared to the signal before injection in group injected with EP-3533. The results from DCE-MRI showed a relatively fast enhancement of normalized signal intensity from the lesion linings after EP-3533 injection in both suture and injection model lesions. This initial signal enhancement can be due to the build-up of the excess probe in the circulation followed by washout. In the suture model, as the unbound probe clears out gradually and decreases the signal intensity, the specific binding of the probe to its target simultaneously increases the signal. Here, we did not observe a significant difference in the signal intensity from 5-min post-injection to 55 min which might be because of the same rate of unbound probe washout and specific binding. This can ultimately create an equilibrium which can be seen as a relatively flat line in the MR signal enhancement plot after the completion of both washout and targeted accumulation. As a result, the signal experiences no further significant change and reaches a plateau. In the injection model as the unbound probe clears out gradually and decreases the signal intensity, the specific binding of the probe to its target simultaneously increases. Here, the unbound probe washout was higher, compared to specific binding which leads to lower final signal enhancement, compared to the suture model. This can be due to the fact that there was a higher fibrotic content in the suture model, compared to the injection model (Fig. 4c and d) and an overall lower number of lesions for MR analysis in the injection model. The large standard deviation of signal enhancement in both models, especially post-probe injection can be attributed to the heterogeneous nature of the lesions in terms of collagen type I content. For example, cystic lesions have more fluid and thin fibrotic wall which can be harder to analyze using MR image analysis software and have a potential to increase error in measurements. While younger lesions can minimize cystic characteristics they are likely to have less fibrotic content. The baboon model of endometriosis with deep nodular lesions, on the other hand, is associated with the highest fibrotic content [62] and could help solve this problem. In our future studies, we plan to utilize this model as it mimics human deep nodular lesions characterized by less cystic features and a higher proportion of fibrotic content [62]. Regardless, quantitative ICP-OES analysis demonstrated significantly higher Gd content in endometriotic lesions in both models after EP-3533 injection, compared to accumulation in muscle tissues or in both lesion and muscle tissues after the injection of EP-3612 non-binding isomer. Histological analysis of harvested endometriotic lesions from these mice showed the presence of a relatively high collagen content, especially in the suture model. This agrees with previous studies showing that collagen content tends to increase in endometriosis over time [41].

The majority of animal models of endometriosis involve the induction of lesions using endometrial fragments that are not properly separated from the surrounding myometrium. Consequently, in some cases, the myometrium constitutes a significant portion of the resulting lesions, which does not accurately reflect the composition of human endometriotic lesions [63]. In both endometriosis models used in this study, the myometrium was included in the endometrial tissue biopsies used for the induction of lesions. However, it was chopped into very fine pieces to produce the lesions. Also, based on Masson’s trichrome-stained images of lesions (Fig. 4), despite the significant presence of collagen in endometriotic lesions, we do not see evidence of distinct myometrial tissue around lesions or collagen within the myometrial tissue and the lack of separation between myometrium and endometrium is unlikely to affect the accumulation of EP-3533 in the lesions.

We need to acknowledge that besides endometriotic lesions, EP-3533 could insignificantly while non-specifically accumulate in other organs, as observed in previous studies [42, 64]. To reduce this effect, other collagen type I binding probes such as CM-101 with much more stable macrocycle gadoterate meglumine (Gd-DOTA) chelate and better pharmacokinetics are being developed and studied [65]. Furthermore, 68Ga-labeled version of EP-3533 is currently being evaluated in clinical trials for type 1 collagen deposition in idiopathic pulmonary fibrosis (NCT05621252), so that clinical translation to the patients with endometriosis could be significantly simplified.

It is also important to note that certain surgeries and laparoscopic procedures may cause adhesions and the development of fibrotic scars [66, 67]. Specific endometriotic lesions, such as cesarean scar endometriosis, may arise due to the presence of scar tissue resulting from prior abdominal surgeries [68, 69] and interfere with EP3533 imaging. For that reason, a patient’s clinical history should be taken into account while differentiating surgically-induced scars from endometriotic lesions, particularly based on the documented anatomical location of previous surgeries [70, 71].

Overall, our data demonstrated for the first time that fibrosis, which is considered a pathological feature of endometriosis, can be further investigated as an appropriate target for EP-3533. After performing initial studies in murine models, we plan to embark on imaging endometriosis in large models such as baboons, with which we have extensive experience and which better recapitulates human disease [36, 55]. Concurrently, we are developing image-guided therapies (theranostics) for endometriosis based on EP-3533 targeting aiming to inhibit signaling pathways that cause the disease [52, 54, 55].

## Conclusion

Considering the consistent presence of fibrosis in all endometriosis disease forms, we consider it an excellent biomarker, which could be used for diagnostic and/or therapeutic delivery purposes. Here, we evaluated a Gd-based collagen type I targeting probe (EP-3533) as an MR imaging probe and showed its utility for detection of endometriotic lesions in mouse models of endometriosis.

### Supplementary Information

ESM 1 (DOCX 13492 kb)

## References

[1] Zondervan KT, Becker CM, Koga K, Missmer SA, Taylor RN, Viganò P (2018). Endometriosis. Nat Rev Dis Primers.

[2] Malvezzi H, Marengo EB, Podgaec S, Piccinato CA (2020). Endometriosis: current challenges in modeling a multifactorial disease of unknown etiology. J Translat Med.

[3] Prentice A (2001). Regular review-endometriosis. British Med J.

[4] Harada T (2013). Dysmenorrhea and endometriosis in young women. Yonago Acta Med.

[5] Gruber TM, Mechsner S (2021). Pathogenesis of endometriosis: the origin of pain and subfertility. Cells.

[6] Surrey E, Soliman AM, Trenz H, Blauer-Peterson C, Sluis A (2020). Impact of endometriosis diagnostic delays on healthcare resource utilization and costs. Adv Ther.

[7] Marinho MC, Magalhaes TF, Fernandes LFC, Augusto KL, Brilhante AV, Bezerra LR (2018). Quality of life in women with endometriosis: an integrative review. J Womens Health.

[8] Missmer SA, Tu FF, Agarwal SK (2021). Impact of endometriosis on life-course potential: a narrative review. Int J Gen Med.

[9] Hosper NA, van den Berg PP, de Rond S (2013). Epithelial-to-mesenchymal transition in fibrosis: collagen type I expression is highly upregulated after EMT, but does not contribute to collagen deposition. Exp Cell Res.

[10] Rolla E (2019) Endometriosis: advances and controversies in classification, pathogenesis, diagnosis, and treatment. F1000Research 8:529 10.12688/f1000research.14817.1 PMC6480968 31069056

[11] Alimi Y, Iwanaga J, Loukas M, Tubbs RS (2018) The clinical anatomy of endometriosis: a review. Cureus 10:e3361 10.7759/cureus.3361 PMC6257623 30510871

[12] Parasar P, Ozcan P, Terry KL (2017). Endometriosis: epidemiology, diagnosis and clinical management. Curr Obstet Gynecol Rep.

[13] Canis M, Donnez JG, Guzick DS (1997). Revised American Society for Reproductive Medicine Classification of Endometriosis: 1996. Fertil Steril.

[14] Lee S-Y, Koo Y-J, Lee D-H (2021). Classification of endometriosis. Yeungnam Univ J Med.

[15] Khazali S (2016). Endometriosis classification-the quest for the Holy Grail?. J Reprod Infertil.

[16] Becker CM, Bokor A, Heikinheimo O (2022). ESHRE guideline: endometriosis. Hum Reprod Open.

[17] Pascoal E, Wessels J, Aas-Eng M (2022). Strengths and limitations of diagnostic tools for endometriosis and relevance in diagnostic test accuracy research. Ultrasound Obstet Gynecol.

[18] Wykes CB, Clark TJ, Khan KS (2004). Accuracy of laparoscopy in the diagnosis of endometriosis: a systematic quantitative review. BJOG.

[19] Nilufer R, Karina B, Paraskevi C et al (2018) Large-scale genome-wide association meta-analysis of endometriosis reveals 13 novel loci and genetically-associated comorbidity with other pain conditions. BioRxiv:406967

[20] May K, Conduit-Hulbert S, Villar J, Kirtley S, Kennedy S, Becker C (2010). Peripheral biomarkers of endometriosis: a systematic review. Hum Reprod Update.

[21] Kiesel L, Sourouni M (2019). Diagnosis of endometriosis in the 21st century. Climacteric.

[22] Nisenblat V, Bossuyt PM, Farquhar C, Johnson N, Hull ML (2016) Imaging modalities for the non-invasive diagnosis of endometriosis. Cochrane Database Syst Rev 2:CD009591 10.1002/14651858.CD009591.pub2 PMC7100540 26919512

[23] Kinkel K, Frei KA, Balleyguier C, Chapron C (2006). Diagnosis of endometriosis with imaging: a review. Eur Radiol.

[24] Chapron C, Marcellin L, Borghese B, Santulli P (2019). Rethinking mechanisms, diagnosis and management of endometriosis. Nat Rev Endocrinol.

[25] Savelli L (2009). Transvaginal sonography for the assessment of ovarian and pelvic endometriosis: how deep is our understanding?. Ultrasound Obstet Gynecol.

[26] Exacoustos C, Manganaro L, Zupi E (2014). Imaging for the evaluation of endometriosis and adenomyosis. Best Pract Res Clin Obstet Gynaecol.

[27] Bourgioti C, Preza O, Panourgias E (2017). MR imaging of endometriosis: spectrum of disease. Diagn Interv Imaging.

[28] Samreen N, Bookwalter CA, Burnett TL (2019). MRI of endometriosis: a comprehensive review. Appl Radiol.

[29] Siegelman ES, Oliver ER (2012). MR imaging of endometriosis: ten imaging pearls. Radiographics.

[30] Zhang H, Li J, Sun W (2014). Hyaluronic acid-modified magnetic iron oxide nanoparticles for MR imaging of surgically induced endometriosis model in rats. PLoS One.

[31] Bianek-Bodzak A, Szurowska E, Sawicki S, Liro M (2013) The importance and perspective of magnetic resonance imaging in the evaluation of endometriosis. BioMed Res Int 2013:436589 10.1155/2013/436589 PMC3854449 24350271

[32] Morawski AM, Lanza GA, Wickline SA (2005). Targeted contrast agents for magnetic resonance imaging and ultrasound. Curr Opin Biotechnol.

[33] Foti PV, Farina R, Palmucci S (2018). Endometriosis: clinical features, MR imaging findings and pathologic correlation. Insights imaging.

[34] Moses AS, Demessie AA, Taratula O, Korzun T, Slayden OD, Taratula O (2021). Nanomedicines for endometriosis: lessons learned from cancer research. Small.

[35] Guo S-W (2014). An overview of the current status of clinical trials on endometriosis: issues and concerns. Fertil Steril.

[36] Zhang Q, Duan J, Olson M, Fazleabas A, Guo S-W (2016). Cellular changes consistent with epithelial–mesenchymal transition and fibroblast-to-myofibroblast transdifferentiation in the progression of experimental endometriosis in baboons. Reprod Sci.

[37] Guo S-W (2018). Fibrogenesis resulting from cyclic bleeding: the Holy Grail of the natural history of ectopic endometrium. Hum Reprod.

[38] Guo S-W, Groothuis PG (2018). Is it time for a paradigm shift in drug research and development in endometriosis/adenomyosis?. Hum Reprod Update.

[39] Vigano P, Candiani M, Monno A, Giacomini E, Vercellini P, Somigliana E (2018). Time to redefine endometriosis including its pro-fibrotic nature. Hum Reprod.

[40] Polasek M, Yang Y, Schühle DT (2017). Molecular MR imaging of fibrosis in a mouse model of pancreatic cancer. Sci Rep.

[41] Zhang Q, Liu X, Guo S-W (2017). Progressive development of endometriosis and its hindrance by anti-platelet treatment in mice with induced endometriosis. Reprod Biomed Online.

[42] Caravan P, Das B, Dumas S (2007). Collagen-targeted MRI contrast agent for molecular imaging of fibrosis. Angew Chem Int Ed.

[43] Helm PA, Caravan P, French BA (2008). Postinfarction myocardial scarring in mice: molecular MR imaging with use of a collagen-targeting contrast agent. Radiology.

[44] Caravan P, Das B, Deng Q et al (2009) A lysine walk to high relaxivity collagen-targeted MRI contrast agents. Chem Commun (4):430–432 10.1039/b819098d 19137175

[45] Li Z, Lu B, Lin J (2021). A Type I collagen-targeted mr imaging probe for staging fibrosis in Crohn’s disease. Front Mol Biosci.

[46] Atanasova I, Sojoodi M, Leitão HS (2020). Molecular MR imaging of fibrin deposition in the liver as an indicator of tissue injury and inflammation. Investig Radiol.

[47] Zhu B, Wei L, Rotile N (2017). Combined magnetic resonance elastography and collagen molecular magnetic resonance imaging accurately stage liver fibrosis in a rat model. Hepatology.

[48] Zhou IY, Catalano OA, Caravan P (2020). Advances in functional and molecular MRI technologies in chronic liver diseases. J Hepatol.

[49] Zhou IY, Clavijo Jordan V, Rotile NJ (2020). Advanced MRI of liver fibrosis and treatment response in a rat model of nonalcoholic steatohepatitis. Radiology.

[50] Polasek M, Fuchs BC, Uppal R (2012). Molecular MR imaging of liver fibrosis: a feasibility study using rat and mouse models. J Hepatol.

[51] Caravan P, Yang Y, Zachariah R (2013). Molecular magnetic resonance imaging of pulmonary fibrosis in mice. Am J Respir Cell Mol Biol.

[52] Moldovan GE, Song Y, Kim TH (2022). Notch effector recombination signal binding protein for immunoglobulin kappa J signaling is required for the initiation of endometrial stromal cell decidualizationdagger. Biol Reprod.

[53] Moldovan GE, Miele L, Fazleabas AT (2021). Notch signaling in reproduction. Trends Endocrinol Metab.

[54] Su RW, Strug MR, Joshi NR (2015). Decreased Notch pathway signaling in the endometrium of women with endometriosis impairs decidualization. J Clin Endocrinol Metab.

[55] Afshar Y, Miele L, Fazleabas AT (2012). Notch1 is regulated by chorionic gonadotropin and progesterone in endometrial stromal cells and modulates decidualization in primates. Endocrinology.

[56] Yoo JY, Kim TH, Shin JH (2022). Loss of MIG-6 results in endometrial progesterone resistance via ERBB2. Nat Commun.

[57] Song Y, Su RW, Joshi NR (2020). Interleukin-6 (IL-6) activates the NOTCH1 signaling pathway through E-proteins in endometriotic lesions. J Clin Endocrinol Metab.

[58] Krieg T, Abraham D, Lafyatis R (2007). Fibrosis in connective tissue disease: the role of the myofibroblast and fibroblast-epithelial cell interactions. Arthritis Res Ther.

[59] Giannandrea M, Parks WC (2014). Diverse functions of matrix metalloproteinases during fibrosis. Dis Model Mech.

[60] Viganò P, Ottolina J, Bartiromo L (2020). Cellular components contributing to fibrosis in endometriosis: a literature review. J Minim Invasive Gynecol.

[61] Tirado-González I, Barrientos G, Tariverdian N (2010). Endometriosis research: animal models for the study of a complex disease. J Reprod Immunol.

[62] Donnez O, Van Langendonckt A, Defrere S (2013). Induction of endometriotic nodules in an experimental baboon model mimicking human deep nodular lesions. Fertil Steril.

[63] Grümmer R (2006). Animal models in endometriosis research. Hum Reprod Update.

[64] Fuchs BC, Wang H, Yang Y (2013). Molecular MRI of collagen to diagnose and stage liver fibrosis. J Hepatol.

[65] Farrar CT, Gale EM, Kennan R (2018). CM-101: type I collagen–targeted MR imaging probe for detection of liver fibrosis. Radiology.

[66] Maciver AH, McCall M, Shapiro AJ (2011). Intra-abdominal adhesions: cellular mechanisms and strategies for prevention. Int J Surg.

[67] Herrick SE, Wilm B (2021). Post-surgical peritoneal scarring and key molecular mechanisms. Biomolecules.

[68] Zhang P, Sun Y, Zhang C (2019). Cesarean scar endometriosis: presentation of 198 cases and literature review. BMC Womens Health.

[69] Xue M, Jackson CJ (2015). Extracellular matrix reorganization during wound healing and its impact on abnormal scarring. Adv Wound Care.

[70] Ten Broek RP, Issa Y, van Santbrink EJ et al (2013) Burden of adhesions in abdominal and pelvic surgery: systematic review and met-analysis. BMJ 347:f5588 10.1136/bmj.f5588 PMC3789584 24092941

[71] Parker MC, Ellis H, Moran BJ (2001). Postoperative adhesions: ten-year follow-up of 12,584 patients undergoing lower abdominal surgery. Dis Colon Rectum.

