# Correction: Detection of Endometriosis Lesions Using Gd-Based Collagen I Targeting Probe in Murine Models of Endometriosis

**DOI:** 10.1007/s11307-023-01848-z

**Published:** 2023-08-17

**Authors:** Nazanin Talebloo, Maria Ariadna Ochoa Bernal, Elizabeth Kenyon, Christiane L. Mallett, Asgerally Fazleabas, Anna Moore

**Affiliations:** 1 Precision Health Program, Michigan State University, 766 Service Road, East Lansing, MI 48824 USA; 2 Department of Chemistry, College of Natural Sciences, Michigan State University, 578 S Shaw Lane, East Lansing, MI 48824 USA; 3 Department of Obstetrics, Gynecology & Reproductive Biology, Michigan State University, 400 Monroe Avenue NW, Grand Rapids, MI 49503 USA; 4 Department of Animal Science, Michigan State University, 474 S Shaw Ln, East Lansing, MI 48824 USA; 5 Department of Radiology, College of Human Medicine, Michigan State University, East Lansing, MI 48824 USA; 6 Institute for Quantitative Health Science and Engineering, Michigan State University, 775 Woodlot Drive, East Lansing, MI 48824 USA

**Correction: Molecular Imaging and Biology**

10.1007/s11307-023-01833-6

The original online version of this article was revised to include the following funding information:

This work was supported in part by the NIH HD 099090 to A.F. and by the Eunice Kennedy Shriver National Institute of Child Health & Human Development of the National Institutes of Health under Award Number T32HD087166, and Michigan State University to M.A.O.B. The content is solely the responsibility of the authors and does not necessarily represent the official views of the National Institutes of Health.

The original article has been corrected.

