# Supplementary material for: Detection of Endometriosis Lesions Using Gd-Based Collagen I Targeting Probe in Murine Models of Endometriosis
Source: Mol Imaging Biol. 2023 Jul 7;25(5):833–43. doi: 10.1007/s11307-023-01833-6 (PMC10598151; doi:10.1007/s11307-023-01833-6)
Supplement: Supplementary file 1 — (DOCX 13492 kb) [file 11307_2023_1833_MOESM1_ESM.docx]

ELECTRONIC SUPPLEMENTARY MATERIAL

**Detection of endometriosis lesions using Gd-based collagen I targeting probe in murine models of endometriosis**

Journal: Molecular Imaging and Biology

Nazanin Talebloo^1,2^, M. Ariadna Ochoa Bernal ^3,4^, Elizabeth Kenyon^1,5^, Christiane Mallet^5,6^, Asgerally Fazleabas^3^, Anna Moore^1,5*^

^1^Precision Health Program, Michigan State University, 766 Service Road, East Lansing, MI 48824

^2^Department of Chemistry, College of Natural Sciences, Michigan State University, 578 S Shaw Lane, East Lansing, MI 48824

^3^Department of Obstetrics, Gynecology & Reproductive Biology, Michigan State University, 400 Monroe Avenue NW, Grand Rapids, MI 49503

^4^Department of Animal Science, Michigan State University, 474 S Shaw Ln #1290, East Lansing, MI 48824, USA

^5^Department of Radiology, College of Human Medicine, Michigan State University, 766 Service Road, East Lansing, MI 48824

^6^Institute for Quantitative Health Science and Engineering, Michigan State University, 775 Woodlot Drive, East Lansing, MI 48824

^*^Corresponding author: Anna Moore, [moorea57@msu.edu](mailto:moorea57@msu.edu)


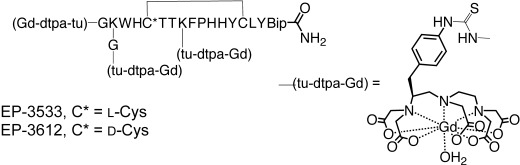


Supplementary Figure 1. Collagen-targeted contrast agents; L-amino acids are designated by one letter code, except where noted; Gd chelates are appended through the N terminus, through branched Lys-Gly residues at the N terminus, and through a Lys side chain within the cyclic portion of the peptide. Caravan, Peter, et al. "Collagen‐targeted MRI contrast agent for molecular imaging of fibrosis." Angewandte Chemie International Edition 46.43 (2007): 8171-8173. Copyright Wiley-VCH GmbH. Reproduced with permission.


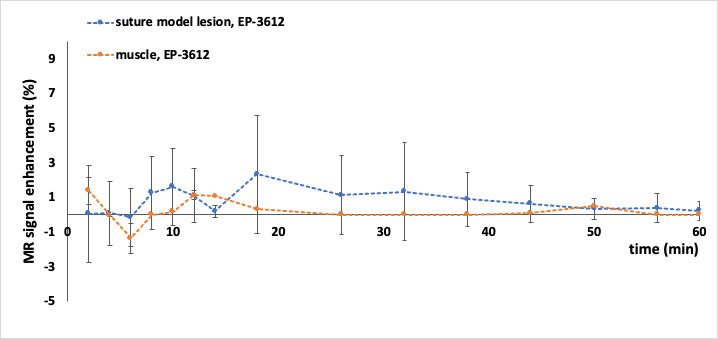


**b**

**a**


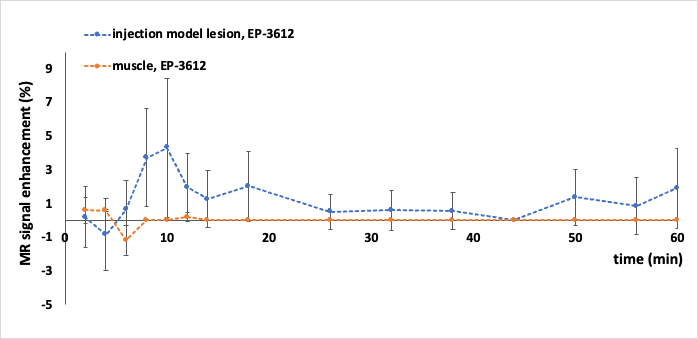


Supplementary Figure 2. Analysis of DCE MRI signal enhancement of endometriotic lesions (blue) and muscle (orange) from mice injected with EP-3612 in a) suture and b) injection models. Data were analyzed as described for the EP-3533 injection.

Supplementary Figure 3. Representative MR images and corresponding fluorescence images in the GFP channel. a, and b) representative images of the suture model of endometriosis from the experimental group (injected with EP-3533); c, and d) representative images of the injection model of endometriosis from the experimental group (injected with EP-3533); e, and f) representative images of the suture model of endometriosis from the control group (injected with EP-3612); g, and h) representative images of the injection model of endometriosis from the control group (injected with EP-3612). Anatomical structures on MR and fluorescence images are depicted for enhanced visualization of the relative locations of endometriotic lesions in relation to surrounding tissues and organs. The images demonstrate the presence of intestines, muscle tissue, bladder, and fat tissue, offering increased clarity of the spatial relationship between the lesions and adjacent anatomical features. Red and green dotted ovals: endometriotic lesions.

**b**

**a**


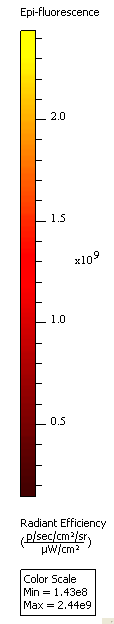

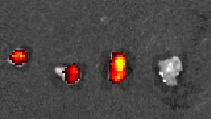


**lesions**

**muscle**


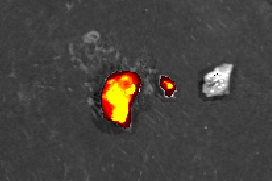


**lesions**

**muscle**

Supplementary Figure 4. Ex vivo fluorescence imaging of excised GFP-expressing endometriotic lesions and control muscle tissues collected from a) suture model and b) injection model.


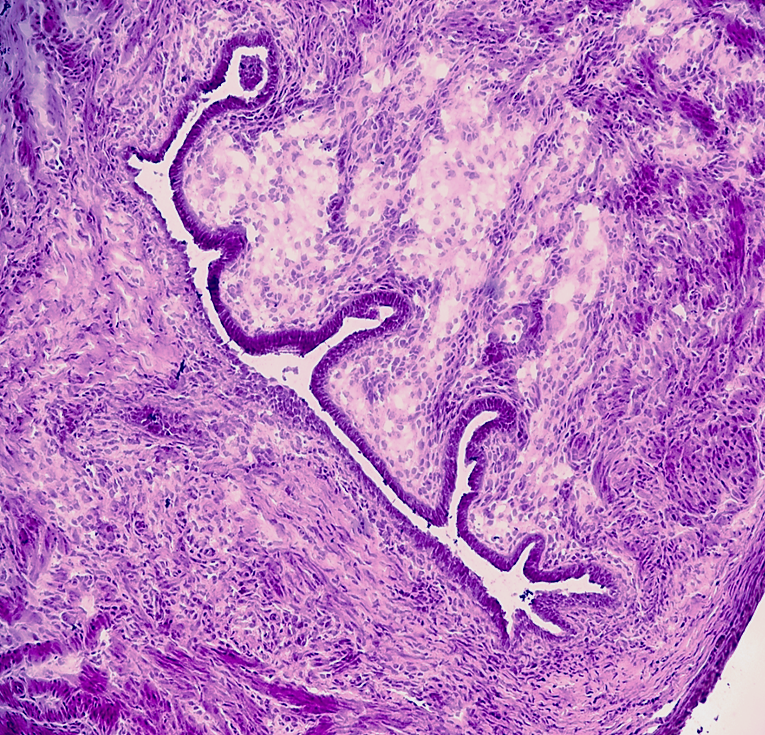

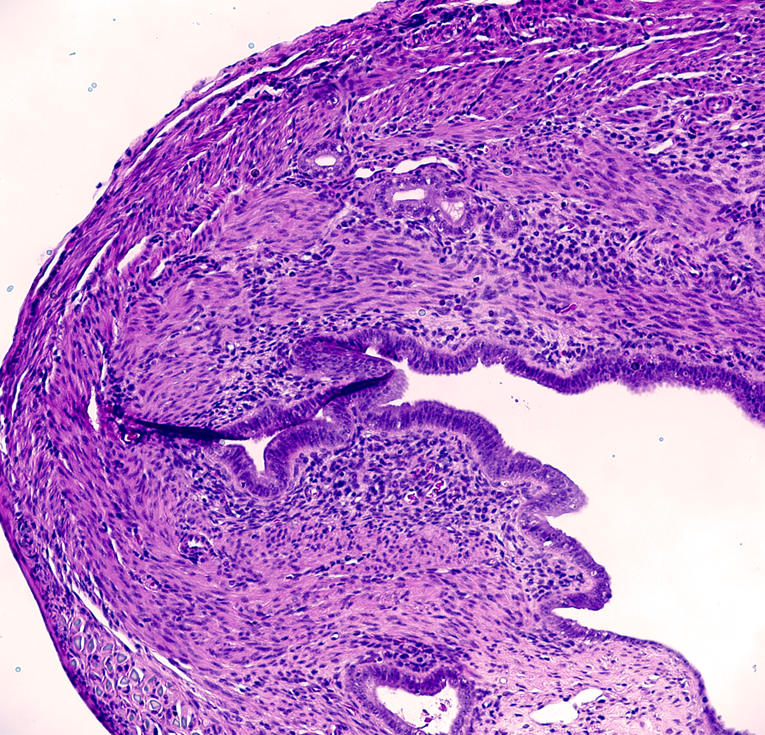


**a**

**b**

**100 um**

**100 um**

Supplementary Figure 5. H&E staining of glands and stroma in the lesions from a) suture and b) injection models. Black arrows: stroma; yellow arrows: glands. Magnification bar =100µm.
